# Mobile Intervention to Improve Sleep and Functional Health of Veterans With Insomnia: Randomized Controlled Trial

**DOI:** 10.2196/29573

**Published:** 2021-12-09

**Authors:** Erin Dawna Reilly, Stephanie A Robinson, Beth Ann Petrakis, Melissa M Gardner, Renda Soylemez Wiener, Carmen Castaneda-Sceppa, Karen S Quigley

**Affiliations:** 1 Mental Illness Research, Education, and Clinical Center VA Bedford Healthcare System Bedford, MA United States; 2 Social and Community Reintegration Research VA Bedford Healthcare System Bedford, MA United States; 3 University of Massachusetts Medical School Worcester, MA United States; 4 Center for Healthcare Outcomes and Implementation Research VA Bedford Healthcare System Bedford, MA United States; 5 Pulmonary Division Boston University School of Medicine Boston, MA United States; 6 William James College Newton, MA United States; 7 Northeastern University Boston, MA United States

**Keywords:** cognitive behavioral therapy, mobile app, physical activity, insomnia

## Abstract

**Background:**

Insomnia is a prevalent and debilitating disorder among veterans. Cognitive behavioral therapy for insomnia (CBTI) can be effective for treating insomnia, although many cannot access this care. Technology-based solutions and lifestyle changes, such as physical activity (PA), offer affordable and accessible self-management alternatives to in-person CBTI.

**Objective:**

This study aims to extend and replicate prior pilot work to examine whether the use of a mobile app for CBTI (cognitive behavioral therapy for insomnia coach app [CBT-i Coach]) improves subjective and objective sleep outcomes. This study also aims to investigate whether the use of the CBT-i Coach app with adjunctive PA improves sleep outcomes more than CBT-i Coach alone.

**Methods:**

A total of 33 veterans (mean age 37.61 years, SD 9.35 years) reporting chronic insomnia were randomized to use either the CBT-i Coach app alone or the CBT-i Coach app with a PA intervention over 6 weeks, with outcome measures of objective and subjective sleep at pre- and posttreatment.

**Results:**

Although the PA manipulation was unsuccessful, both groups of veterans using the CBT-i Coach app showed significant improvement from baseline to postintervention on insomnia (*P*<.001), sleep quality (*P*<.001), and functional sleep outcomes (*P*=.002). Improvements in subjective sleep outcomes were similar in those with and without posttraumatic stress disorder and mild-to-moderate sleep apnea. We also observed a significant but modest increase in objective sleep efficiency (*P*=.02).

**Conclusions:**

These findings suggest that the use of a mobile app–delivered CBTI is feasible and beneficial for improving sleep outcomes in veterans with insomnia, including those with comorbid conditions such as posttraumatic stress disorder or mild-to-moderate sleep apnea.

**Trial Registration:**

ClinicalTrials.gov NCT03305354; https://clinicaltrials.gov/ct2/show/NCT03305354

## Introduction

### Background

Sleep disturbances, especially chronic insomnia (difficulty falling and staying asleep), are among the most prevalent complaints following military deployments, particularly in post-9/11 veterans of Operations Enduring Freedom, Iraqi Freedom, and New Dawn [1-4]. According to an evaluation of medical records of approximately 10 million US veterans, 91% were prescribed at least one sleep medication, with sleep apnea (47%) and insomnia (26%) being two of the most common diagnoses [5]. There are both service-related and psychological reasons why sleep disturbances can emerge during deployment and endure past active military service. These include unusual sleep–wake schedules because of military work schedules, separation from family and loved ones, poor sleeping conditions that are detrimental to good sleep hygiene, and threat of or actual injury to self or others and associated subjective arousal [6,7]. Moreover, military service can have additional repercussions for postdeployment reintegration into civilian life [8], including greater risk for specific psychiatric comorbidities of sleep disturbance such as posttraumatic stress disorder (PTSD) [9], depression [10], and chronic pain [11].

The co-occurrence of mental and physical symptoms with sleep disturbance can add complexity to this health issue. For instance, evidence now suggests that sleep disturbance can precede or follow the onset of depression, suggesting that in some cases, sleep disturbance can increase the likelihood of depressive symptoms [12-14], including in veterans [10]. It is also possible that both sleep disturbance and depression are different manifestations of an underlying metabolic or energy regulatory dysfunction, as it has been theorized that energy regulation is the key function of all nervous systems [15,16].

Regardless of the root cause, sleep disturbance in post-9/11 veterans also commonly co-occurs with poor functional health and reduced social and community engagement [17-19]. Chronic insomnia is a transdiagnostic health problem affecting physical, cognitive, and emotional functioning [4,20], which in turn can complicate veteran community reintegration (CR) or the ability to return to family, vocational, and community life [21]. Research on insomnia suggests that poor sleep can negatively affect specific areas of CR, such as academic functioning issues [22], employment success [23], and social relationships related to loneliness and feelings of belonging [24]. Poor functioning and CR are a particularly serious and common problem for veterans with mental health concerns who frequently do not themselves seek out or avail health care [25,26]. This further affects the difficulty of treating poor sleep, as the confluence of insomnia and comorbid mental health concerns in veterans together can negatively affect treatment efficacy for sleep outcomes [27,28].

### Cognitive Behavioral Therapy for Insomnia

The use of over-the-counter sleep aids and prescription medications is common and remains a frontline treatment for insomnia [29]. However, meta-analytic research is mixed, with some showing that pharmacological and nonpharmacological insomnia interventions have comparable efficacy [30] and others suggesting that behavioral interventions show greater improvements in sleep quality than pharmacological interventions [31]. The effects of nonpharmacological interventions tend to be more durable than sleep medications, with treatment gains persisting after treatment [32].

Consequently, cognitive behavioral therapy for insomnia (CBTI) is considered the gold standard nonpharmacological treatment for insomnia in terms of both effectiveness and efficacy [32-34]. This manualized treatment implements multiple cognitive and behavioral techniques, including sleep restriction, psychoeducation, and cognitive restructuring, and has been evaluated as a frontline intervention among veterans [35,36]. However, for veterans facing competing demands from work, school, family, and other health concerns, adherence to in-clinic CBTI treatment can be poor [37]. A major barrier to in-person CBTI treatment is its time-intensive nature, which reduces both accessibility and adherence [7].

Mobile apps delivering self-management–based, personalized CBTI have the potential to reduce these barriers and assist with in-person treatments [38-40]. To capitalize on this delivery option, the cognitive behavioral therapy for insomnia coach app (CBT-i Coach app; US Department of Veterans Affairs) was launched in 2013 [41]. Although the app was not intended to completely replace CBTI provider–delivered interventions, it is a valuable adjunct to provide sleep-management tools and educational resources and, in particular, to track self-reported sleep metrics and subjective sleep. Although existing research is preliminary, the CBT-i Coach app has shown promise in positively affecting sleep when used in coordination with individual CBTI treatments [42] and as a stand-alone self-management option [43].

In addition to preliminary, positive research findings on self-management of insomnia via mobile sleep apps, interest in physical activity (PA) for insomnia treatment has also increased as clinicians look for conjunctive behavioral interventions for sleep improvement. Often, treatment as usual for insomnia includes a health care provider’s recommendation for improving lifestyle choices such as eating a healthier diet and increasing exercise as a way to promote better sleep [7]. Such routine clinician suggestions for increasing PA are based on research studies suggesting that taking more daily steps is positively associated with both longer sleep duration and better sleep quality at night [44]. Even modest increases in PA have been proposed to provide an easily accessible, nonpharmacological treatment alternative for sleep disturbance [45]. Specifically, some research suggests that long-term use of moderate aerobic exercise can improve sleep quality and functioning in individuals with insomnia [46] and obstructive sleep apnea (OSA) [47]. However, a recent meta-analysis found both positive and negative associations between daily PA and sleep duration [48], suggesting that other factors such as insomnia severity and mental health comorbidities may affect the direction of an often-assumed positive relationship between exercise and sleep. Given the common recommendation for increased PA in patients with insomnia, these findings suggest that future research is needed to actively explore what impact, if any, PA can have for patients already using a common insomnia self-management program.

### This Study

In a recent pilot project, we used a combination of the CBT-i Coach app and a home-based objective sleep monitor to assess and provide feedback to post-9/11 veterans regarding their home-based sleep behaviors [40]. The CBT-i Coach app had high usability, and veterans with insomnia showed improvements in both self-reported and sleep monitor–assessed sleep quality. This study also revealed that a large proportion of these veterans (54%) unexpectedly screened positive for OSA using the WatchPAT (Itamar Medical, Inc) at-home sleep monitor, a sleep monitor validated for detecting OSA [49]. As we excluded individuals for whom either of the 2 initial home-based sleep tests suggested greater than mild sleep apnea, this pilot was unfortunately unable to characterize the impact of the mobile sleep intervention on their insomnia. In addition, prior research has suggested that a PA adjunct could potentially provide additional benefits to sleep outcomes in veterans, even for those with mild-to-moderate sleep apnea.

This project seeks to replicate initial pilot results on sleep outcomes, while extending prior work to include veterans with mild-to-moderate sleep apnea. We also assess the outcomes of PA as an adjunctive treatment in a second intervention group that used the CBT-i Coach app and guide them to increase their PA via increased daily steps (PA). Thus, we conduct a small single-blind, pilot randomized controlled trial (RCT; randomized 1:1) with the following 2 arms: (1) CBT-i Coach app use only (CBT-i only) and (2) CBT-i Coach app use plus a PA intervention (CBT-i +PA). We examine the following a priori, preregistered Clinical Trials.gov (identifier: NCT03305354) for sleep hypotheses and analyses:

1. The group using the CBT-i Coach app for 6 weeks, with an additional PA intervention component (CBT-i +PA), would report better subjective sleep outcomes (self-reported insomnia severity, sleep quality, and functional sleep) and objective sleep outcomes (sleep efficiency) compared with participants using only the CBT-i Coach app.
2. There would be pre- to postintervention improvements in subjective sleep outcomes, objective sleep efficiency, and functional measures for all participants (both CBT-i +PA and CBT-i only).

In addition, we examine 2 a priori registered hypotheses related to functional health and community engagement:

1. There would be improvements in self-reported community integration and functioning outcomes over the course of the trial for each group and for the full sample.
2. There would be improvements in subjective sleep as reported by participants in the CBT-i Coach app sleep diaries for total sleep, sleep efficiency, and self-reported sleep quality, for each group and for the full sample.

## Methods

### Participant Recruitment

**Figure 1:**
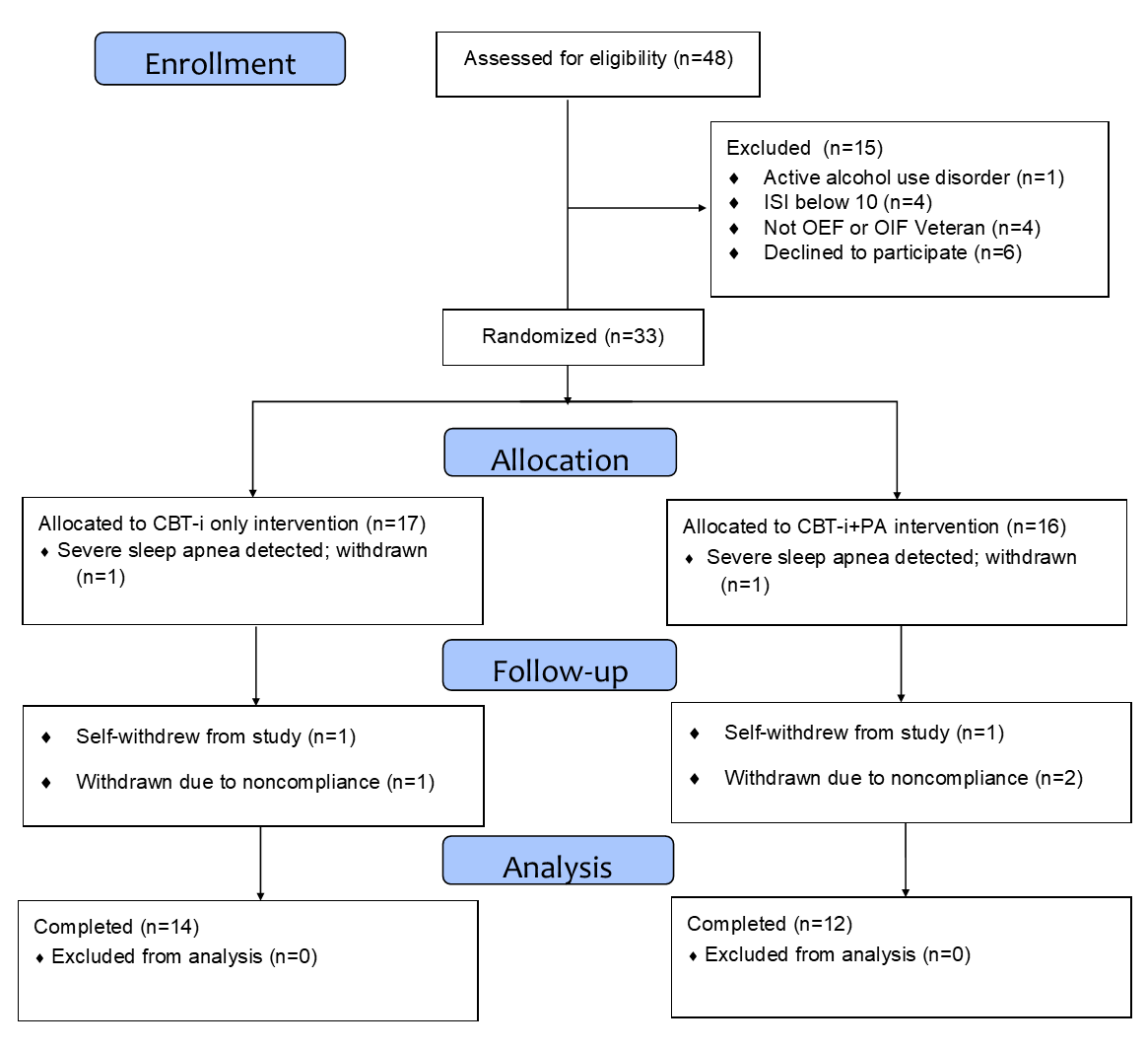
CONSORT diagram of participant inclusion and attrition. CBT-i: cognitive behavioral therapy for insomnia; ISI: insomnia severity index; OEF: Operation Enduring Freedom; OIF: Operation Iraqi Freedom; PA: physical activity.

The study was approved by the institutional review board of the VA Bedford Healthcare System, Bedford, Massachusetts. Veterans were recruited via flyers, presentations, community outreach, provider referrals, and recruitment letters. Interested veterans were phone screened for study eligibility. The participant flow has been described in Figure 1.

### Participant Demographics

The mean age of the randomized sample was 37.61 years (SD 9.35 years), and the mean BMI was 28.03 (SD 4.27; ie, overweight). Participants (25/33, 76% men and 8/33, 24% women) had an average baseline insomnia severity index (ISI) of 17.91 (SD 4.05), indicating moderately severe clinical insomnia. Approximately 76% (25/33) identified as White and 18% (6/33) identified ethnically as Hispanic. No participants had been diagnosed with apnea before enrollment; however, the WatchPAT sleep assessment (completed by 32/33, 97% of the enrolled participants) revealed that 38% (12/32) had an apnea–hypopnea index (AHI) score corresponding to no or minimal apnea, 25% (8/32) had mild apnea, 31% (10/32) had moderate apnea, and 6% (2/32) had severe apnea. In addition, none of the participants had previously used the CBT-i Coach app (0/33, 0% of participants), and only some (4/33, 12%) participants had previously used the Fitbit (Fitbit Inc) tracker and mobile app (see Table 1 for detailed demographic information).

**Table 1:** Descriptive statistics at baseline for participants by group (N=33).

Characteristics		CBT-i Coach only group (n=17)	CBT-i Coach+physical activity (n=16)	Total
Age (years), mean (SD)		36.24 (8.74)	39.06 (10.00)	37.61 (9.35)
BMI (kg/m^2^), mean (SD)		26.84 (4.43)	29.30 (4.12)	28.03 (4.27)
Baseline ISI^a^, mean (SD)		18.00 (3.57)	17.81 (4.57)	17.91 (4.05)
Baseline weekly steps, mean (SD)		9487 (5628)	9942 (4841)	9697.14 (5181)
**Gender, n (%)**				
	Male	12 (71)	13 (81)	25 (76)
	Female	5 (29)	3 (19)	8 (24)
**Apnea rates^b^, n (%)**				
	None or minimal	5 (31)	7 (44)	12 (38)
	Mild	4 (25)	4 (25)	8 (25)
	Moderate	6 (38)	4 (25)	10 (31)
	Severe	1 (6)	1 (6)	2 (6)
**Race^b^, n (%)**				
	White/Caucasian	13 (76)	12 (75)	25 (76)
	Black/African American	2 (12)	3 (19)	5 (15)
	Japanese	1 (6)	0 (0)	1 (3)
	Middle Eastern	1 (6)	0 (0)	1 (3)
	American Indian	1 (6)	0 (0)	1 (3)
	Other	2 (12)	1 (6)	3 (9)
**Ethnicity, n (%)**				
	Hispanic/Latino	4 (24)	2 (13)	6 (18)
	Not Hispanic/Latino	13 (76)	14 (87)	27 (82)
**Income (US $)^b^, n (%)**				
	<11,999	2 (12)	1 (7)	3 (9)
	12,000-24,999	3 (18)	0 (0)	3 (9)
	25,000-49,999	7 (41)	4 (27)	11 (34)
	50,000-99,000	1 (6)	3 (20)	4 (13)
	≥100,000	4 (24)	7 (47)	11 (34)

^a^ISI: insomnia severity index.

^b^Not required to answer or missing data point; numbers may not add up to the total sample of N=33.

### Description of the Interventions: CBT-i Coach App, Self-management Guide, Fitbit, and WatchPAT

#### Overview

For 6 weeks, all participants were asked to use the CBT-i Coach app, to which we added self-management support. Participants were randomized to either (1) use of the CBT-i Coach app alone (CBT-i alone) or (2) CBT-i Coach plus PA intervention (CBT-i+PA). Those in the CBT-i +PA group were asked to use the CBT-i Coach app and increase their average daily step count by 10% each week or until they walked 10,000 steps a day. All participants were loaned a Fitbit Charge 2, WatchPAT, and iPod Touch on which the CBT-i Coach app was installed (iOS version 2.3). Participants wore the WatchPAT 3 times: (1) one night soon after study enrollment; (2) a second time, generally within the first week after enrollment; and (3) one night after they had completed the 6-week intervention. On average, the time between participants’ first and last WatchPAT data collection night was 44 days.

#### CBT-i Coach App

The CBT-i Coach app offers sleep psychoeducation, tools for tracking sleep (eg, daily sleep diaries), and sleep hygiene recommendations, including cultivating a conducive sleep environment, engaging in regular exercise, and maintaining a healthy diet. In-app relaxation tools include guided imagery audio clips, tips for winding down, breathing tools, and progressive muscle relaxation. A behavioral plan can also be reviewed and updated in the CBT-i Coach app, including setting reminders for when to go to sleep and get out of bed, completing sleep diaries, taking insomnia assessments, engaging in scheduled worry time, and stopping caffeine intake for the day. The CBT-i Coach app allows users to see graphical depictions of their sleep diary data and ISI scores. Additional CBTI worksheets were embedded in a separate in-house–created app (Sleep Help app) and included wakeful activities, coping self-statements, constructive worry, and a relaxation log similar to worksheets from the workbook *Quiet Your Mind and Get To Sleep* [50]. The daily input of a sleep diary required approximately 2 to 3 minutes per day to complete. The amount of time that individual participants allotted to additional CBT-i Coach app activities that were suggested (but not required) for participants via the self-management guide (see the following section) varied. We estimated that these required an additional 3 (eg, completing a sleep assessment) to 6 minutes (eg, progressive muscle relaxation meditation).

#### Self-management Guide

A self-management guide (Multimedia Appendix 1; CBT-i +PA group version), available on both the iPod Touch and paper copy, provided week-by-week suggestions for using elements of the app and the worksheets. For each week of the 6-week intervention, the guide suggested what materials to read in the app; which features to use, such as completing a daily sleep diary each morning; and which worksheets to complete. Participants in the CBT-i +PA arm were provided with additional instructions, guidelines, and resources to help motivate and support their weekly step increases. Specifically, their version of the self-management guide reminded these participants to increase their daily step counts every week, reminded them to track their steps daily in their paper log, and requested that they *boost* their step counts by 10% weekly. They were also provided with a 24-page researcher-modified motivational and instructional guide titled *Stepping Out*, which was based on prior research, clinical guidelines, and national walking guidebooks (ie, *Stepping Out Mature Adults: Be Healthy, Walk Safely* [51]). Completion of these PA-specific tasks added approximately 8 to 10 minutes per week to their activities. This PA intervention was purposefully *light touch* and involved gentle motivational reminders, simulating common real-world clinical suggestions given to patients and allowed us to assess the feasibility of a PA adjunct for a possible future implementation trial.

#### WatchPAT Sleep Monitor

Objective sleep was recorded using a WatchPAT (model WP200U) sleep monitor (Itamar Medical, Inc). The WatchPAT sleep monitor is a Food and Drug Administration–approved device that assesses objective sleep parameters and is valid for screening measures of OSA [49] and broad sleep stage measures (eg, rapid eye movement [REM] vs non-REM) as compared with polysomnography-based measures [52]. The WatchPAT is worn like a wristwatch attached via a cable to a plethysmographic-based finger-mounted probe and a small sensor on the chest to measure snoring. It is less obtrusive and less disruptive of sleep than in-laboratory or in-home polysomnography. Participants can use the device with simple instructions, which were provided via a video on the iPod Touch and a laminated pamphlet. Participants also self-recorded any prescription medications and nonprescription medications, including vitamins taken during the 24 hours before each WatchPAT night.

#### PA Monitor

PA was recorded with a Fitbit Charge 2, a wireless-enabled tool that provides measures of heart rate, quality of sleep, number of steps walked, steps climbed, and estimates calories burned. It is worn like a wristwatch and has been well-validated for use in measuring steps [53].

### Measures

#### Overview

Participants had to have served in the military since 2001 in Iraq or Afghanistan and report current insomnia defined by an ISI score>10 with a duration of at least 1 month and impaired daytime functioning as measured by endorsing *much* or *very much* on ISI item 7. This ISI cutoff was chosen from prior research, which found that a total score of ≥11 on the ISI indicated insomnia disorder in clinical samples with 97.2% sensitivity and 100% specificity [54]. As some research has suggested that insomnia is underdiagnosed in the veteran population [5], participants were not required to have a formal, prior diagnosis of insomnia disorder within their electronic medical records. Participants were excluded if they demonstrated moderate-to-severe cognitive impairment on the Telephone Mini Mental State Exam [55] and excessive alcohol use on the Alcohol Use Disorders Identification Test-Concise [56] or reported periodic leg movement symptoms or a circadian rhythm disorder. As subjective and objective measures of sleep often do not correlate highly in patients with insomnia [57] and can differentially predict treatment outcomes [54], we followed best practices and measured the impact of our intervention on both subjective and objective sleep outcomes.

#### Subjective Sleep Measurement

Self-reports of insomnia, sleep quality, and functional outcomes because of sleep were measured at baseline and final assessment visits using the ISI, the Pittsburgh Sleep Quality Index (PSQI), and the Functional Outcomes of Sleep Questionnaire–10 items (FOSQ-10), respectively. The ISI has been shown to be sensitive to changes in insomnia severity with CBTI interventions [58,59], with possible scores ranging from 0 to 28 and higher scores indicating more severe insomnia. The PSQI, a global measure of perceived sleep quality, has been extensively used in sleep trials and has been shown to be sensitive to changes after CBTI [37,59]. Scores on the PSQI range from 0 to 21, with a higher score indicating worse sleep quality. The FOSQ-10 [60] was used to assess the impact of sleepiness on functioning in everyday activities. Possible scores range from 5 to 20, with higher scores indicating a better functional status. In this sample, Cronbach α were acceptable (ISI, Cronbach α=.70; PSQI, Cronbach α=.75; and FOSQ-10, Cronbach α=.82). Sleep diary data were collected through the CBT-i Coach app and included self-reported total sleep time, sleep quality (1=very poor to 5=very good), and app-calculated sleep efficiency (percentage of time spent asleep while in bed).

#### Objective Sleep Measurement

Objectively measured sleep efficiency was obtained from the WatchPAT across 3 nights. To control for first night effects, we used the second WatchPAT sleep assessment night as the preintervention sleep efficiency (on average, approximately 8 days after enrollment). The WatchPAT calculates the proportion of REM sleep using a genetic algorithm (ie, a machine learning technique) to determine REM sleep onset and offset. The WatchPAT recorded total sleep time, total and percent time spent in light, deep and REM sleep stages, AHI, respiratory disturbance index, and number of awakenings; for this study, sleep efficacy was the primary measure of interest.

#### Functioning and Mental Health Outcomes

Additional outcomes included social engagement, community engagement, and health-related quality of life. The Lubben Social Network Scale–6 items (LSNS-6) [61] is a 6-item scale measuring 5 aspects of social isolation and social networks, with scores ranging from 0 to 30 and higher scores indicating lower levels of social isolation. The Community Integration Questionnaire (CIQ) [62] is a 15-item self-report measure that assesses productivity and engagement in activities in the home and in social settings, with a range of 0 to 39 points and greater scores indicating greater community engagement. Finally, the Veterans RAND 12 Item Health Survey (VR-12) [63] is a reliable and effective measure that documents perceived change in veterans’ health-related quality of life. It provides both a mental component score (MCS) and a physical component score (PCS) ranging from 0 to 100, with higher scores indicating better functioning. The LSNS-6 (α=.87) and VR-12 (α=.94) showed good internal reliability. The CIQ showed poor internal reliability in this sample (α=.40), and as such, CIQ results should be interpreted with caution.

Additional measures were used to assess whether our randomization process resulted in balanced groups in terms of mental health and physical symptoms. These were evaluated using the Patient Health Questionnaire (PHQ)-15 [64], depressive symptoms using the PHQ-9 [65] and pain disability index [66], and PTSD symptoms with the PTSD checklist (PCL-5) [67]. Each of these measures demonstrated good internal consistency (PHQ-15: α=.83; PHQ-9: α=.82; pain disability index: α=.82; and PCL-5: α=.95).

### Procedure

After a brief phone screen to assess for potential eligibility based on veteran status, age, and insomnia severity, participants were scheduled for an initial study visit to complete the consent process, eligibility testing, and baseline study activities. At the initial study visit, participants were provided with an informed consent document detailing the study expectations, procedures, requirements for participation (including use of different technologies such as the CBT-i Coach app, Fitbit, and WatchPAT), assessment schedule, and compensation. The informed consent document and Health Insurance Portability and Accountability Act authorization form were also reviewed verbally by a research staff member (BAP, EDR, or SAR) before being signed by the participant. The potential participants were then provided with an opportunity to ask any questions. Only after all questions were answered did the participants sign the written consent to participate and complete self-report measures and surveys. Participants were then randomly assigned to CBT-i alone or CBT-i +PA intervention. A researcher demonstrated the use of the iPod Touch, CBT-i Coach app, Fitbit, WatchPAT, and Sleep Help app. All participants were instructed to complete daily sleep diaries, complete activities in the self-management guide, and wear the WatchPAT once during the next week. A follow-up visit was scheduled for the participant to return after wearing the WatchPAT to assess whether participants screened positive for severe apnea. Individuals randomized to the CBT-i +PA condition were given a step-tracking document, an adapted handbook for safely increasing one’s steps [68], and instructions to increase their step count by 10% each week over the 6 weeks.

During the second visit, a researcher downloaded and printed the data from the WatchPAT sleep report. Individuals with no apnea diagnosis or a positive screen for mild or moderate OSA (AHI<30/hour of sleep) at either the first or second WatchPAT testing were retained in the trial, and those with severe OSA (AHI>30/hour of sleep) were excluded from further participation and referred to a primary care provider. Given the well-known *first night effect* in which sleep can be negatively affected by sleep monitoring [69], particularly in those with insomnia [70], participants then completed a second night of WatchPAT monitoring within the coming week, which was reviewed again. At the third and final in-person visit (ie, postintervention), participants returned all devices, completed a postintervention survey battery and interview, and received their final WatchPAT report. Participants received US $15 for the 2 in-person assessment screenings during preintervention, US $15 for a midintervention phone assessment, and US $400 for the postintervention assessment.

### Power Analysis

To meet our original pilot goal of ultimately calculating a reliable effect size for a later fully-powered RCT, we conducted a priori power analyses for the proposed nonparametric analyses (using GPower 3.1 [71]). For an effect size Cohen f=0.3 (moderate via Cohen conventions; α=.05, 1-β=.80, 2 groups with 5 potential covariates), we would need N=45 for each of the 2 groups. For this pilot RCT, we proposed that N=15 per group would provide a reasonable estimate of the effect sizes for each group for the future RCT, as numerous such studies appear in the peer-reviewed literature with similar group numbers (eg, group N=10, 11, or 17 [72])

### Data Analysis Plan

We first assessed the potential differences between groups on sleep, functioning, and mental health outcomes. In addition, we completed a planned manipulation check to ensure that participants in the CBTI+PA group increased their steps relative to the CBTI alone group. To address our a priori hypotheses, we evaluated whether subjective (ISI, PSQI, and FOSQ-10) and objective sleep efficiency changed from preintervention to postintervention using analyses suitable for our small sample size, namely Wilcoxon signed rank tests and Mann–Whitney *U* tests comparing the medians of distributions. To address our functioning and mental health hypotheses, we used Wilcoxon signed rank tests to assess changes between baseline and postintervention on the LSNS-6, CIQ, and VR-12 MCS, and PCS scores. Outcomes were analyzed using an intent-to-treat approach, which included all randomized participants. To account for missing data, we used a *last observation called forward* method so we could use the last completed follow-up as outcome data for all participants not removed for meeting exclusion criteria (eg, severe apnea) or initial protocol noncompliance (eg, not completing the full baseline procedures for intervention onboarding).

## Results

### Preliminary Analyses

Nonparametric Wilcoxon signed rank tests revealed no significant group differences between the CBTI alone and CBT-i+PA groups on baseline subjective and objective sleep measures (ISI, PSQI, FOSQ-10, and sleep efficiency). The daily use of the CBT-i Coach app was high; participants on average completed sleep diaries for 86% (36/42 days) of study nights. In addition, there were no significant between-group differences in baseline mental and physical health on the VR-12. However, importantly, groups differed significantly at baseline on the PCL-5 (*P*=.03, with a median score of 20.50 for the CBTI alone group and a median score of 42.50 for the CBTi+PA group. Thus, post hoc analyses were conducted to assess the potential impact of the differences in PTSD symptoms on sleep outcomes.

Manipulation check analyses revealed that there were no significant differences between baseline steps (mean 10,193, SD 6161) and postintervention steps (mean 8382, SD 4629) for the CBTI alone group (Z=−0.80; *P*=.41) and no significant differences between baseline steps (mean 9485, SD 4798) and postintervention steps (mean 7653, SD 3941) for the CBT-i+PA group (Z=−1.96; *P*=.05). In addition, the CBT-i+PA group did not significantly increase their steps compared with the CBTI alone group (Z=−0.92; *P*=.37), indicating a failure of the PA manipulation.

### Sleep Outcomes

There were no significant differences between the CBT-i alone group and the CBT-i+PA group from preintervention to postintervention for the ISI (*U*=66; *P*=.37), PSQI (*U*=76; *P*=.98), FOSQ-10 (*U*=77.5; *P*=.74), or sleep efficiency (*U*=54; *P*=.60; see Table 2 for mean, median, and SD descriptive information by group).

Across all participants using the CBT-i Coach app (ie, without considering group and per a priori hypothesis 2), there was a significant decrease in ISI scores (Z=−4.31; *P*<.001) from preintervention (median 16.00) to postintervention (median 11.00), which reflects a decrease from moderate clinical insomnia (ISI range 15-21) to subthreshold insomnia (ISI range 8-14), although the 5-point median decrease was below the suggested 6-point cutoff for clinical improvement. Of the 26 final participants, 24 (92%) reported reduced insomnia symptoms over the 6-week intervention, and 2 (8%) reported increased insomnia symptoms. There was also a significant decrease in PSQI scores (Z=−3.57; *P*<.001) from preintervention (median 14.00) to postintervention (median 9.00), with 77% (20/26) of participants reporting improved sleep quality, 15% (4/26) reporting their sleep as worse, and 8% (2/26) reporting no difference. The median PSQI score at postintervention (median 9.00) fell below the suggested cutoff for clinically significant insomnia for veterans (cutoff=10).

There was also a significant increase in FOSQ-10 scores (Z=3.13; *P*=.002) from preintervention (median 14.00) to postintervention (median 15.58), with 77% (20/26) of participants reporting better functional sleep outcomes and 23% (6/26) reporting worse functioning. The median improvement of 1.58 points was slightly below the suggested clinically significant minimal important difference (1.7-2.0), although this minimal important difference is suggested for participants with greater insomnia severity because of narcolepsy or diagnosed OSA. Given the high rate of sleep apnea, we also ran a post hoc analysis to investigate the impact of baseline apnea on changes in sleep outcomes. Those without apnea reported significant improvement in their ISI (Z=−2.807; *P*=.005), as did participants with mild to moderate sleep apnea (*Z=*−3.19; *P*<.001), suggesting that mild to moderate apnea did not prevent intervention-related ISI improvement.

Using available WatchPAT data (n=23), we observed a significant increase in sleep efficiency (*Z*=−2.4; *P*=.02) from preintervention (median 84.13) to postintervention (median 85.32). Specifically, 65% (15/23) of participants showed an increase in sleep efficiency over the 6-week intervention from the second WatchPAT monitor assessment to the last, whereas 35% (8/23) showed a reduction in overall sleep efficiency. In addition, post hoc Friedman tests for nonparametric repeated measures were used to compare total sleep times, sleep efficiency, and sleep quality reported in the CBT-i Coach app sleep diary across the 6 weeks of the trial for the full sample (CBT-i and CBT-i +PA). Using this self-reported data, there were no significant differences across weekly averages for total sleep (*χ*^2^_5,22_=2.3; *P*=.81) or sleep efficiency (*χ*^2^_5,22_=9.3; *P*=.10). However, there was a significant increase in reported sleep quality across the 6 weeks (*χ*^2^_5,22_=12.6; *P*=.03), with post hoc Bonferroni-adjusted tests revealing a significant increase in sleep quality from week 1 (median 2.64) to week 5 (median 3.14; *P*=.03).

**Table 2:** Outcome measures at preintervention and postintervention.

Measures	Scale range	CBT-i^a^ only group (n=14)					*P* value		CBT-i+physical activity group (n=12)						*P* value
		Preintervention		Postintervention				Preintervention			Postintervention				
		Values, mean (SD)	Values, median (range)	Values, mean (SD)	Values, median (range)			Values, mean (SD)		Values, median (range)	Values, mean (SD)	Values, median (range)			
ISI^b^	0-28	17.64 (3.77)	16.00 (13.00-26.00)	10.86 (4.24)	11.00 (3.00-22.00)	<.001		17.92 (4.94)		17.00 (12.00-26.00)	13.08 (5.09)	13.50 (4.00-23.00)	.004		
PSQI^c^	0-21	13.07 (3.29)	13.50 (8.00-19.00)	9.86 (4.24)	9.50 (3.00-18.00)	.004		12.63 (4.52)		14.00 (4.00-18.00)	10.50 (4.56)	9.50 (2.00-19.00)	.03		
FOSQ-10^d^	5-20	13.67 (3.50)	14.33 (5.83-19.00)	15.57 (3.27)	16.25 (9.50-19.33)	.04		12.67 (3.22)		12.00 (7.67-17.17)	14.90 (2.53)	15.44 (10.33-18.50)	.02		
LSNS-6^e^	0-30	11.64 (7.40)	11.50 (1.00-21.00)	11.71 (7.13)	12.00 (2.00-22.00)	.84		9.42 (4.36)		9.00 (1.00-19.00)	10.00 (4.45)	10.00 (3.00-19.00)	.52		
CIQ^f^	0-39	28.43 (3.00)	29.00 (22.00-32.00)	27.57 (3.98)	28.00 (21.00-33.00)	.19		27.42 (3.42)		28.00 (21.00-32.00)	29.00 (3.57)	29.50 (23.00-34.00)	.15		
VR-12^g^ PCS^h^	0-100	44.70 (9.04)	43.74 (22.34-56.61)	43.44 (9.06)	43.43 (24.31-56.85)	.30		41.81 (11.32)		41.06 (23.67-57.19)	40.85 (8.59)	40.85 (26.36-54.46)	.75		
VR-12 MCS^i^	0-100	38.04 (13.73)	38.84 (18.87-56.61)	44.05 (13.63)	40.26 (26.54-64.35)	.02		39.97 (10.93)		39.11 (19.48-58.32)	38.49 (14.60)	39.75 (14.37-62.97)	.53		

^a^CBT-i: cognitive behavioral therapy for insomnia coach app.

^b^ISI: insomnia severity index.

^c^PSQI: Pittsburgh Sleep Quality Index.

^d^FOSQ-10: Functional Outcomes of Sleep Questionnaire–10.

^e^LSNS-6: Lubben Social Network Scale–6 items.

^f^CIQ: Community Integration Questionnaire.

^g^VR-12: Veterans RAND 12 Item Health Survey.

^h^PCS: physical component score.

^i^MCS: mental component score.

### Functioning and Mental Health Outcomes

Mann–Whitney *U* tests revealed no significant differences between groups in changes from preintervention to postintervention for the LSNS-6 (*U*=75; *P*=.67), the VR-12 PCS (*U*=78; *P*=.78), or CIQ (*U*=46; *P*=.05). However, there was a significant between-group difference in the VR-12 MCS (*U*=44; *P*=.04). Specifically, the CBT-i +PA group had a statistically significant but numerically small and clinically marginal decrease in VR-12 MCS scores from preintervention (median 40.2) to postintervention (median 39.7; see Table 2 for descriptive information by group). Across groups, there were no significant improvements in the LSNS-6 (*P*=.54), CIQ (*P*=.88), VR-12-PCS (*P*=.15), or VR-12 MCS (*P*=.17).

### Additional Post Hoc Analyses of PTSD and Sleep Outcomes

As preliminary analyses revealed a failure of randomization such that by random chance, there was significantly higher PTSD symptomology in the CBT-i + PA group, we conducted post hoc analyses to evaluate the potential impact of PTSD symptom severity on primary sleep outcomes. Compared with participants below the symptom cutoff suggesting no clinically relevant PTSD symptomology, individuals with PTSD symptom severity in the clinical range (as indicated by a score>33 on the PCL-5) had significantly worse subjective sleep pre intervention (Table 3), as reflected in their baseline ISI (*Z*=−1.99; *P*=.04), PSQI (*Z*=−2.18; *P*=.03), and FOSQ-10 (*Z*=−2.74; *P*=.01). However, those with and without clinically significant PTSD symptoms showed significant improvements from preintervention to postintervention on self-reported sleep outcomes. Specifically, veterans with PTSD symptoms in the clinical range reported significant improvements in their ISI (*Z*=−2.76; *P*=.01), FOSQ-10 (*Z*=−2.40; *P*=.02), and PSQI (*Z*=−2.19; *P*=.03) scores, as did the group without clinically significant PTSD symptom severity on the ISI (*Z*=−3.21; *P*<.001), FOSQ-10 (*Z*=−2.29; *P*=.02), and PSQI (*Z*=−2.92; *P*=.01). Using the PCL-5 clinical cutoff scores, we also compared participants with and without clinically significant PTSD symptom severity in terms of their improvement on the ISI (*Z*=1.17; *P*=.43), FOSQ-10 (*Z*=−1.42; *P*=.43), and PSQI (*Z*=0.76; *P*=.43) and found no statistically significant differences.

**Table 3:** Mental health symptom measures at preintervention and postintervention (N=26).

Measures	Scale range	CBT-i^a^ only group (n=14)					CBT-i+physical activity group (n=12)				
		Preintervention		Postintervention		Preintervention				Postintervention	
		Values, mean (SD)	Values, median (range)	Values, mean (SD)	Values, median (range)	Values, mean (SD)		Values, median (range)	Values, mean (SD)		Values, median (range)
PCL-5^b^	0-80	24.21 (17.55)	20.00 (3.00-51.00)	19.43 (13.50)	13.50 (0.00-57.00)	39.50 (20.21)		42.50 (10.00-61.00)	38.08 (22.14)		40.00 (8.00-76.00)
PHQ^c^-15	0-30	9.57 (5.06)	8.00 (4.00-22.00)	7.86 (5.02)	6.50 (1.00-18.00)	11.67 (5.61)		10.50 (5.00-21.00)	10.42 (5.48)		10.50 (2.00-19.00)
PHQ-9	0-27	9.00 (4.22)	10.50 (2.00-14.00)	7.21 (5.41)	5.50 (0.00-18.00)	11.75 (5.31)		11.50 (5.00-22.00)	10.67 (5.57)		9.50 (2.00-21.00)
PDI^d^	0-70	17.43 (12.36)	18.50 (0.00-37.00)	16.71 (15.86)	11.50 (0.00-51.00)	24.42 (13.02)		24.50 (2.00-47.00)	23.33 (14.42)		25.00 (1.00-48.00)

^a^CBT-i: cognitive behavioral therapy for insomnia coach app.

^b^PCL-5: posttraumatic stress disorder checklist–5 items.

^c^PHQ: Patient Health Questionnaire.

^d^PDI: pain disability index.

## Discussion

### Principal Findings

We conducted an RCT with 2 arms (CBT-i Coach app and CBT-i Coach app use plus PA) in veterans reporting chronic insomnia to (1) extend our previous pilot findings to a larger and more inclusive group of veterans and (2) examine the additional effect of an adjunctive PA intervention. Although PA manipulation was not effective, this study provides further evidence of the positive impact of the CBT-i Coach app for veterans with insomnia. We replicated our prior CBT-i Coach app findings [40], observing significant improvement from preintervention to postintervention in self-reported sleep quality and functional outcomes related to sleep, this time in a larger and more heterogeneous veteran sample. Participants also reported significant improvement in the ISI, although the median change of 5 points was just below the suggested clinically significant 6-point reduction [73]. Objective sleep efficiency data from the WatchPAT device also revealed a small but significant improvement in sleep efficiency. This concordance of improvement in both subjective and objective sleep measures is notable, as past research has not observed consistent results across subjective and objective sleep measures [74]. This supports the conclusion that self-management–based use of the CBT-i Coach app can improve multiple indicators of sleep quality, quantity, efficiency, and functioning.

### Insomnia and PTSD Findings

Despite randomization, veterans in the CBT-i +PA arm had significantly higher rates of PTSD. Those with PTSD symptomology that fell above a cutoff suggestive of clinical severity reported significantly worse insomnia at baseline. This association between PTSD and insomnia is partially reflected in the diagnostic criteria for PTSD, which includes nightmares and sleep disturbances [75], and thus may be because of somatic PTSD symptoms similar to or associated with symptoms of insomnia. Alternatively, veterans with PTSD often experience nightmares and heightened alertness to somatic sensations that can negatively affect their ability to stay asleep.

Interestingly, participants with and without clinical-level PTSD symptom severity had significantly and similarly improved sleep outcomes after 6 weeks of using the CBT-i Coach app. Prior work has shown that CBTI is an efficacious treatment for individuals with comorbid PTSD [59]; however, no prior work has examined this efficacy for app-delivered CBTI. This finding is of practical importance, as a recent survey revealed that some veterans with PTSD prefer treatments focusing on insomnia rather than on PTSD and may also prefer mobile or remote-delivered self-management treatment for insomnia [76]. Mobile-delivered insomnia treatments may additionally provide an entryway to interventions for those who are initially unwilling or unable to access in-person PTSD or other mental health treatments. Future work is needed to disambiguate the potential condition or symptom overlap of PTSD and insomnia and the impact of the CBT-i Coach app on comorbid insomnia and PTSD symptoms.

### Sleep Apnea and CBTI Mobile Intervention

Our results also emphasize the importance of including veterans with mild or moderate sleep apnea when evaluating behavioral interventions for insomnia. In prior work, we excluded participants with greater than mild sleep apnea, as we were concerned that the CBT-i Coach app may not be effective when the probable etiology of the sleep disturbance was OSA. Although here we excluded participants with severe sleep apnea, the broader inclusion criteria in this study led to a sample where almost one-third (10/32, 31%) had mild-to-moderate sleep apnea. These participants also benefited from the mobile intervention, with significant improvement in insomnia severity and sleep quality at par with those without apnea. This finding suggests that a mobile CBTI intervention can still benefit veterans with mild-to-moderate sleep apnea.

### Limitations and Future Directions

Several study limitations must be considered. First, this study used a small sample (n<20 per group) and a single site. Thus, the findings may not be representative of a larger population of veterans with insomnia. Future studies with larger samples will enable the use of parametric analyses and potential covariates (eg, sex and BMI). Second, our PA intervention did not result in the needed step increase that would have enabled us to effectively test our between-group hypotheses. This manipulation failure may have been because of high preintervention steps for the PA group, which had a baseline mean step count of 9942 (SD 4841), only a few steps away from the 10,000-step goal typically given in PA interventions (ie, a ceiling effect) [77]. The CBT-i Coach app also includes some recommendations for engaging in regular exercise. Thus, it is possible that the non-PA group may have also increased their step count, although they were given self-management instructions that did not mention the PA components and did not receive the walking guide or step-tracking motivational sheet provided to the intervention group. Future research should examine the potential adjunctive role of increased PA in a group that is more sedentary and typical of young to middle-aged US samples.

Finally, because of the failure of PA manipulation, we must be cautious in interpreting outcomes related to the use of the CBT-i Coach app. Owing to our manipulation failure (ie, that participants did not increase their PA through our self-management program), our planned methodology for a pilot, single-blind, 2-group randomized controlled study lacked an effective control group. We also lacked a usual care or an additional intervention group, which would have allowed for a more robust analysis of the CBT-i Coach app’s impact on sleep and functioning. Future research should include a control group that does not use the CBT-i Coach app for more robust comparison testing. We attempted to address this issue by using previously validated clinically significant improvement cutoffs for the ISI, PSQI, and FOSQ-10 to allow for more justifiable conclusions regarding the effect sizes of the within-participant sleep improvements.

### Conclusions

This study found significant improvements in subjective and objective sleep across all veterans with chronic insomnia who used mobile-delivered CBTI for 6 weeks. In a group instructed to increase their PA and that had greater PTSD symptom severity, no significant improvements in PA were observed. However, these participants instructed to increase their PA were also more physically active at baseline than expected (ie, greater baseline step counts than typical community participants) and thus had relatively less room for improvement. Importantly, we observed improved sleep outcomes in participants with clinically significant PTSD symptoms and in those with mild-to-moderate sleep apnea after app use. This suggests that interventions using mobile-delivered CBTI can be a feasible and efficacious self-management–based insomnia intervention for many veterans, including those with comorbid conditions such as PTSD and those with mild-to-moderate sleep apnea.

## Appendix

Multimedia Appendix 1 Mobile self-management guide for cognitive behavioral therapy for insomnia+physical activity group.

Multimedia Appendix 2 CONSORT-eHEALTH checklist (V 1.6.1).

## Abbreviations

AHI: apnea–hypopnea index
CBT-i Coach: cognitive behavioral therapy for insomnia coach app
CBTI: cognitive behavioral therapy for insomnia
CIQ: Community Integration Questionnaire
CR: community reintegration
FOSQ-10: Functional Outcomes of Sleep Questionnaire–10 items
ISI: insomnia severity index
LSNS-6: Lubben Social Network Scale–6 items
MCS: mental component score
OSA: obstructive sleep apnea
PA: physical activity
PCL-5: posttraumatic stress disorder checklist–5
PCS: physical component score
PHQ: Patient Health Questionnaire
PSQI: Pittsburgh Sleep Quality Index
PTSD: posttraumatic stress disorder
RCT: randomized controlled trial
REM: rapid eye movement
VR-12: Veterans RAND 12 Item Health Survey
