# Supplementary material for: Mobile Intervention to Improve Sleep and Functional Health of Veterans With Insomnia: Randomized Controlled Trial
Source: JMIR Form Res. 2021 Dec 9;5(12):e29573. doi: 10.2196/29573 (PMC8704109; doi:10.2196/29573)
Supplement: Multimedia Appendix 1 [file formative_v5i12e29573_app1.pdf]

Getting Started

Day 1

To Do	Where?	How?
☐ Check Insomnia Symptoms		My Sleep > Assessments > Take Assessment Now
☐ What you need to know about Insomnia		Learn > Sleep 101> Why Do We Sleep, Sleep Regulators **AND** What is CBT-i?
☐ What you need to know about sleep habits		Learn > Habits & Sleep > Using the Bedroom for Two Activities Only **AND** Watching the Clock
☐ Complete Worksheet on Wakeful Activities		Wakeful Activities

Every Day

To Do	Where?	How?
☐ Complete a sleep diary every morning		My Sleep > Sleep Diary
☐ Track your steps		Wear Fitbit during the day
☐ Record your daily steps		"Tracking Your Walking With Your Fitbit – The 10% Boost Program" > Baseline

Last Day of Week

To Do	Where?	How?
☐ Calculate what your average daily steps were, and your goal for the next week to increase that average by 10%		"Tracking Your Walking With Your Fitbit – The 10% Boost Program" > Baseline (Week 1) > "Weekly Total" through "New Goal"
☐ Ensure that you are walking safely		Start reading through the "Stepping Out" Walking Guide
☐ Coping Self-Statements worksheet		Coping Self-Statements
☐ Improve your Sleep Hygiene		Tools > Create New Sleep habits > Explore the topics here to learn more

Key: CBT-I Sleep Help Fitbit Folder

WEEK 2 SELF-MANAGEMENT GUIDE

Beginning To Improve Sleep Quality

Every Day

To Do	Where?	How?
☐ Complete a sleep diary every morning	CBT-i	My Sleep > Sleep Diary
☐ Track your steps		Wear Fitbit during the day
☐ Aim to walk more throughout the day to meet your new daily step-count goal		
☐ Record your daily steps		"Tracking Your Walking With Your Fitbit – The 10% Boost Program" > Week 2

This Week

To Do	Where?	How?
☐ View 1st sleep prescription with personal recommended bed/wake time.	CBT-i	Sleep Prescription > Continue > Choose Desired Wake Time > Next > Accept<br><br>(<u>Note</u>: You won't be able to set this until you have entered 5 days worth of sleep diaries).
☐ If you wish, set a reminder to tell you when it's your prescribed bed/wake time	CBT-i	Reminders > Prescribed Bed Time/Wake Time
☐ Check your insomnia symptoms	CBT-i	My Sleep > Assessments > Take Assessment Now
☐ Learn about changing your sleep habits	CBT-i	Tools > Create New Sleep Habits - Choose one or two of these each day to learn about or work on
☐ Relax your body	CBT-i	Tools > Quiet Your Mind > Choose a Relaxation Option such as Winding Down, Breathing Tool, Progressive Muscle Relaxation or a Guided Imagery
☐ Re-assess how you are doing by re-doing your Wakeful Activities worksheet		Wakeful Activities
☐ Set up a relaxation goal for the week		Relaxation Log
☐ If you haven't finished reading Stepping Out" then please do it this week		

Last Day of Week

To Do	Where?	How?
☐ Calculate your average daily steps, and your goal for the next week to increase that average by 10%		"Tracking Your Walking With Your Fitbit – The 10% Boost Program" > Week 2 > "Weekly Total" through "New Goal"

Key: CBT-i CBT-I Sleep Help Fitbit Folder

Keeping Up the Hard Work

Every Day

To Do	Where?	How?
☐ Complete a sleep diary every morning		My Sleep > Sleep Diary
☐ Track your steps		Wear Fitbit during the day
☐ Aim to walk more throughout the day to meet your new daily step-count goal		
☐ Record your daily steps		"Tracking Your Walking With Your Fitbit – The 10% Boost Program" > Week 3

This Week

To Do	Where?	How?
☐ Update your recommended bedtime and wake time		Sleep Prescription > Continue > Enter Wake Time > Go through questionnaire > Submit
☐ Check your insomnia symptoms		My Sleep > Assessments > Take Assessment Now
☐ Relax your body		Tools > Quiet Your Mind > Choose a Relaxation Option such as Winding Down, Breathing Tool, Progressive Muscle Relaxation or a Guided Imagery
☐ Set up a new relaxation goal for the week		Relaxation Log
☐ Create a behavioral plan		Behavioral Plan

Last Day of Week

To Do	Where?	How?
☐ Calculate your average daily steps, and your goal for the next week to increase that average by 10%		"Tracking Your Walking With Your Fitbit – The 10% Boost Program" > Week 3 > "Weekly Total" through "New Goal"

Key: CBT-I Sleep Help Fitbit Folder

WEEK 4 SELF-MANAGEMENT GUIDE

Sticking With It

Every Day

To Do	Where?	How?
☐ Complete a sleep diary every morning	CBT-i	My Sleep > Sleep Diary
☐ Track your steps		Wear Fitbit during the day
☐ Aim to walk more throughout the day to meet your new daily step-count goal		
☐ Record your daily steps		"Tracking Your Walking With Your Fitbit – The 10% Boost Program" > Week 4

This Week

To Do	Where?	How?
☐ Check your insomnia symptoms	CBT-i	My Sleep > Assessments > Take Assessment Now
☐ Relax your body	CBT-i	Tools > Quiet Your Mind > Choose a Relaxation Option such as Winding Down, Breathing Tool, Progressive Muscle Relaxation or a Guided Imagery
☐ Learn about how worry can disrupt your sleep	CBT-i	Learn > Habits & Sleep > Worrying in Bed
☐ Learn about how you can change your perspective by changing your thoughts	CBT-i	Tools > Quiet Your Mind > Change Your Perspective
☐ Create a plan for Constructive Worrying	CBT-i	CBT-i> Quiet Your Mind > Schedule Worry Time<br>**OR**<br>Sleep Help > Constructive Worrying
☐ Set up a new relaxation goal for the week		Relaxation Log
☐ Assess how you are doing with your Behavioral Plan		Behavioral Plan and then indicate for which days in the past week you met your goals

Last Day of Week

To Do	Where?	How?
☐ Calculate your average daily steps, and your goal for the next week to increase that average by 10%		"Tracking Your Walking With Your Fitbit – The 10% Boost Program" > Week 4 > "Weekly Total" through "New Goal"

Key: CBT-i CBT-I Sleep Help Fitbit Folder

WEEK 5 SELF-MANAGEMENT GUIDE

Maintaining Gains & Preventing Insomnia (2 weeks to go!)

Every Day

To Do	Where?	How?
☐ Complete a sleep diary every morning	CBT-i	My Sleep > Sleep Diary
☐ Track your steps	Fitbit	Wear Fitbit during the day
☐ Aim to walk more throughout the day to meet your new daily step-count goal		
☐ Record your daily steps	Folder	"Tracking Your Walking With Your Fitbit – The 10% Boost Program" > Week 5

This Week

To Do	Where?	How?
☐ Check your insomnia symptoms	CBT-i	My Sleep > Assessments > Take Assessment Now
☐ Relax your body	CBT-i	Tools > Quiet Your Mind > Choose a Relaxation Option such as Winding Down, Breathing Tool, Progressive Muscle Relaxation or a Guided Imagery
☐ Prevent insomnia in the future. Use this checklist to see if there are any areas where you are having trouble making changes and to see where you might need to concentrate your efforts or make a different kind of change.	CBT-i	Tools > Prevent Insomnia In The Future
☐ Assess how your Constructive Worrying went for the last week. If there are new concerns, add those to your list and remove any that have been resolved (Sleep Help). If you set up a Worry Time, see if you need to revise your concerns or worries (CBT-i).	Sleep Help<br>CBT-i	Sleep Help > Constructive Worrying<br>**OR**<br>CBT-i> Quiet Your Mind > Schedule Worry Time
☐ Set up a new relaxation goal for the week	Sleep Help	Relaxation Log
☐ Assess how you are doing with your Behavioral Plan	Sleep Help	Behavioral Plan, then indicate for which days in the past week you met your goals

Last Day of Week

To Do	Where?	How?
☐ Calculate your average daily steps, and your goal for the next week to increase that average by 10%	Folder	"Tracking Your Walking With Your Fitbit – The 10% Boost Program" > Week 5 > "Weekly Total" through "New Goal"

Key: CBT-I Sleep Help Fitbit Folder

WEEK 6 SELF-MANAGEMENT GUIDE

Maintaining Healthy Physical Activity and Sleep (Last week!)

Every Day

To Do	Where?	How?
☐ Complete a sleep diary every morning		My Sleep > Sleep Diary
☐ Track your steps		Wear Fitbit during the day
☐ Aim to walk more throughout the day to meet your new daily step-count goal		
☐ Record your daily steps		"Tracking Your Walking With Your Fitbit – The 10% Boost Program" > Week 6

This Week

To Do	Where?	How?
☐ Check your insomnia symptoms		My Sleep > Assessments > Take Assessment Now
☐ Relax your body		Tools > Quiet Your Mind > Choose a Relaxation Option such as Winding Down, Breathing Tool, Progressive Muscle Relaxation or a Guided Imagery
☐ Assess how your Constructive Worrying went for the last week. If there are new concerns, add those to your list and remove any that have been resolved (Sleep Help). If you set up a Worry Time, see if you need to revise your concerns or worries (CBT-i).		Sleep Help > Constructive Worrying<br>**OR**<br>CBT-i> Quiet Your Mind > Schedule Worry Time
☐ Set up a new relaxation goal for the week		Relaxation Log
☐ Assess how you are doing with your Behavioral Plan		Behavioral Plan, then indicate for which days in the past week you met your goals

Key: CBT-I Sleep Help Fitbit Folder
